# Role of miR29c in goose fatty liver is mediated by its target genes that are involved in energy homeostasis and cell growth

**DOI:** 10.1186/s12917-018-1653-3

**Published:** 2018-11-06

**Authors:** Long Liu, Qian Wang, Qianqian Wang, Xing Zhao, Pan Zhao, Tuoyu Geng, Daoqing Gong

**Affiliations:** College of Animal Science and Technology, Yangzhou University, Yangzhou, 225009 China

**Keywords:** Fatty liver, Goose, miR29c, *RFX1*, *Insig1*, *Sgk1*, *Col3a1*

## Abstract

**Background:**

A short period of overfeeding can lead to severe hepatic steatosis in the goose, which is physiological, suggesting that geese, as a descendent of a migrating ancestor, may have evolutionally developed a unique mechanism that operates in contrast to the mechanism underlying pathological fatty liver in humans or other mammals. In this study, we report that suppression of miR29c and upregulation of its target genes in goose fatty liver vs. normal liver could be part of a unique mechanism that contributes to the regulation of energy homeostasis and cell growth.

**Results:**

Our data showed that miR29c expression was comprehensively inhibited in energy homeostasis-related tissues (the liver, fat and muscle) of overfed vs. normally fed geese, which is different from miR29c induction that occurs in tissues of the diabetic rat. To address the function of miR29c, three predicted target genes (i.e., *Insig1, Sgk1* and *Col3a1*) that participate in energy homeostasis or cell growth were validated by a dual-fluorescence reporter system and other in vitro assays. Importantly, expression of *Insig1, Sgk1 and Col3a1* was upregulated in goose fatty liver. In line with these observations, treatment of goose hepatocytes with high glucose or palmitate suppressed the expression of miR29c but induced the expression of the target genes, suggesting that hyperglycemia and hyperlipidemia, at least partially, contribute to the suppression of miR29c and induction of the target genes in goose fatty liver. In addition, pharmacological assays indicated that *RFX1* was a transcription factor involved in the expression of miR29c.

**Conclusions:**

This study suggests that miR29c may play a role in the regulation of energy homeostasis and tissue growth via its target genes, contributing to the tolerance of the goose to severe hepatic steatosis.

**Electronic supplementary material:**

The online version of this article (10.1186/s12917-018-1653-3) contains supplementary material, which is available to authorized users.

## Background

Overfeeding, which leads to an energy surplus, is traditionally used as a method to produce goose fatty liver (*foie gras*). In the Landes goose (a liver-specific breed), overfeeding for 3–4 weeks can result in 1000 to 1200 g of fatty liver, which is generally 8–10 times heavier than the normal liver [1]. In the fatty liver, fats account for more than 50% of the composition [1]. Although the liver suffers from severe steatosis, it seldom displays overt pathological symptoms. Several lines of evidence indicate that goose fatty liver displays unique features, including suppressed expression of the *Tnfα* gene (a marker of inflammation) and *Grp78* gene (a marker of endoplasmic reticulum stress) and increased expression of mitochondria-related genes and adiponectin receptors [2–4].

MicroRNAs are a class of small noncoding RNAs (18–25 nucleotides in length) that are largely conserved among species. MicroRNAs usually function by destabilizing transcription or inhibiting translation via binding to the 3′-untranslated region (3’-UTR) of their target genes. The miR29 family, which includes three paralogs (miR29a, miR29b, and miR29c), has multiple cellular functions. Previous studies indicated that the miR29 family inhibits cancer cell migration and invasion by directly targeting LOXL2 in several cancers (e.g., renal cell carcinoma, cervical cell carcinoma, and lung, head and neck squamous cell carcinoma) [5]. In addition to the role of the miR29 family in cancer development, evidence indicates that the miR29 family participates in the regulation of glucose homeostasis. For example, He et al. reported that miR29a/b/c is induced in the skeletal muscles, fatty tissues and liver of diabetic Goto-Kakizaki vs. healthy rats and that adenovirus-mediated overexpression of miR29a/b/c in 3 T3-L1 adipocytes represses insulin-stimulated glucose uptake, contributing to insulin resistance [6].

Our previous study identified 151 microRNAs (miRNAs) that were differentially expressed (114 miRNAs upregulated and 37 miRNAs downregulated) in the liver of overfed geese vs. normally fed geese, suggesting the miRNAs may play an important role in the development of goose fatty liver [7]. miRNA-29c (miR29c) was one of the downregulated genes. However, it is unclear what genes are targeted by miR29c or how it functions in the goose in the development of fatty liver. Studies utilizing mammalian animals/cells have shown that the upregulation of miR29c is positively associated with diabetes, hyperglycemia, and other metabolic disorders [6, 8, 9], in which insulin resistance and loss of glucose homeostasis play a pivotal role [10]. We thus hypothesized that miR29c could regulate glucose/energy metabolism via its target genes in the development of goose fatty liver. To test this hypothesis, the expression of miR29c in energy metabolism-related tissues of the goose and its target genes was determined, and the regulation of miR29c by fatty liver-related factors and transcription factor(s) was analyzed. The findings from this study suggest that miR29c may provide geese tolerance against severe hepatic steatosis by modulating energy homeostasis and tissue growth and that its function is mediated by its target genes, *Col3a1*, *Sgk1*, and *Insig1*.

## Results

### Expression of miR29c was suppressed in energy homeostasis-related tissues of overfed geese

**Fig. 1:**
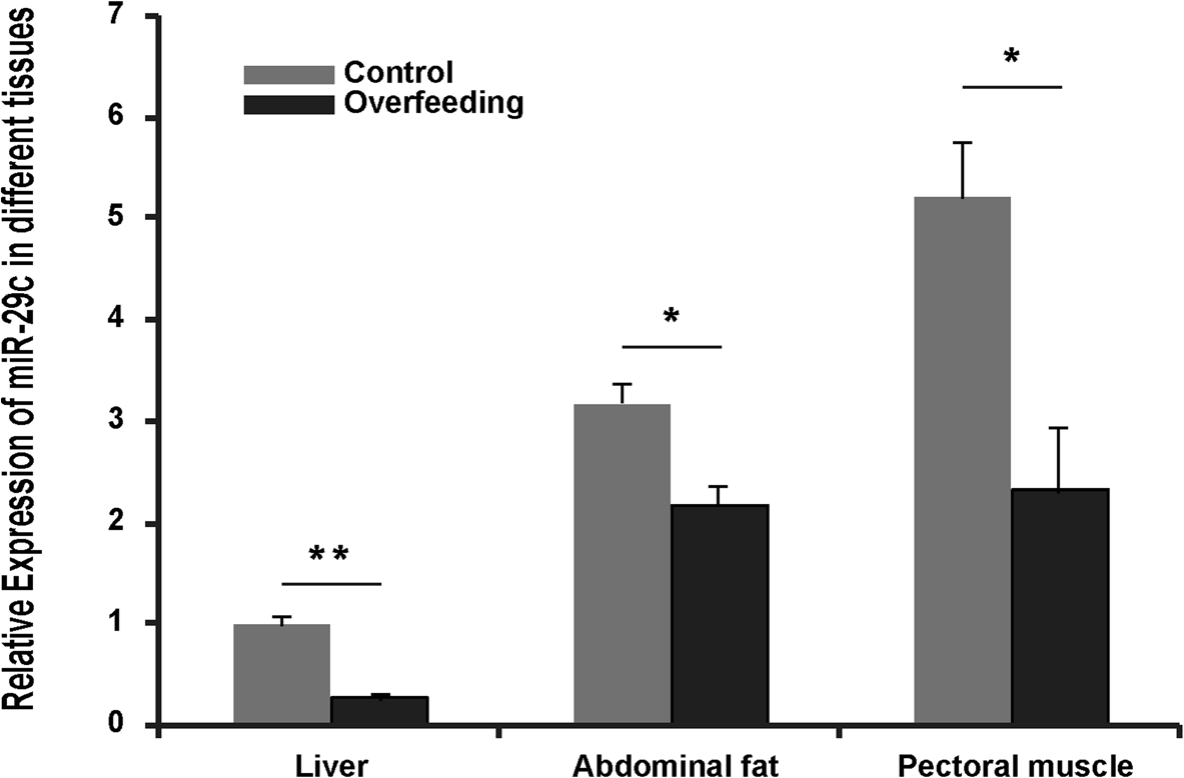
Goose miR29c was comprehensively suppressed in energy metabolism-related tissues of overfed geese vs. normal geese. The expression level of miR29c is presented as the fold change over the control, *n* = 5. *, ** denote *p* < 0.05, 0.01 vs. control, respectively. All of the data are shown as the means ± SEM

To test whether miR29c was involved in the development of goose fatty liver, 70-day-old Landes geese were normally fed or overfed a corn-based diet for 19 days. Liver weight, body weight, the ratio of liver weight to body weight, and liver color all indicated that fatty livers were successfully developed in overfed geese after 19 days of overfeeding, while control geese (i.e., normally fed ones) had normal livers [7]. We performed quantitative PCR analysis on liver samples from geese. The results showed that, compared to the normal liver, the expression of miR29c was significantly suppressed in goose fatty liver (Fig. 1). Similarly, miR29c expression was also inhibited in abdominal fat and pectoral muscle (Fig. 1). The comprehensive suppression of miR29c in energy homeostasis-related tissues of overfed geese suggested that miR29c could have a role in the regulation of energy homeostasis in goose fatty liver.

### Suppression of miR29c was partially attributed to hyperglycemia and hyperlipidemia

**Fig. 2:**
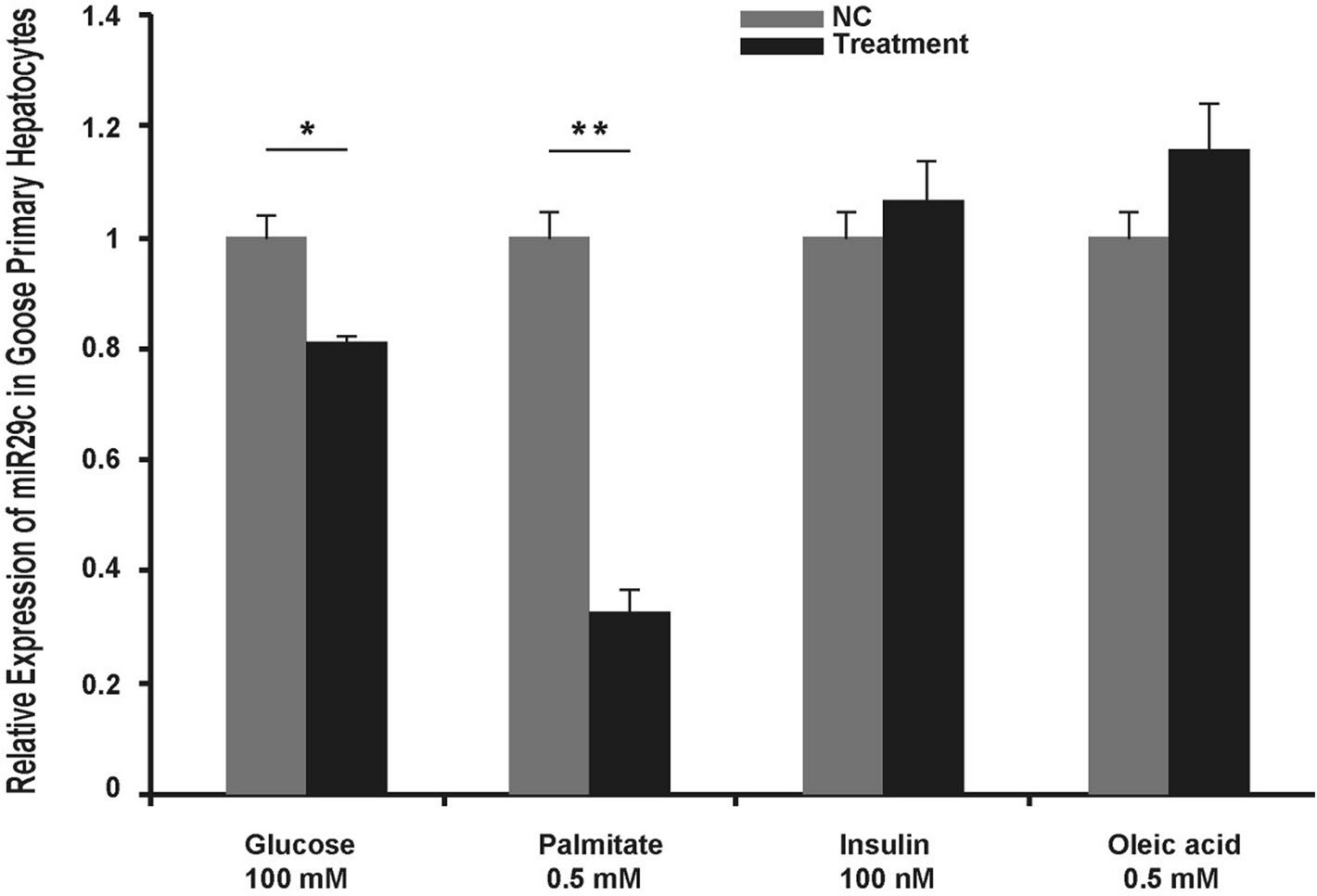
Fatty liver-related factors differentially regulated the expression of miR29c in goose primary hepatocytes. Glucose and palmitate but not insulin or oleic acid suppressed miR29c expression in goose primary hepatocytes. The expression level of miR29c is presented as the fold change over the control, *n* = 4. *, ** denote *p* < 0.05, 0.01 vs. control, respectively. All of the data are shown as the means ± SEM

Compared to control geese, the levels of glucose, insulin and fatty acids (both saturated fatty acids and unsaturated fatty acids) were elevated in the blood of overfed geese [11, 12], and thus, it is possible that suppression of miR29c in the liver of overfed geese was due to some of these factors. To confirm this hypothesis, goose primary hepatocytes were treated with or without glucose, insulin, palmitic acid (a primary saturated fatty acid) and oleic acid (a primary unsaturated fatty acid). Quantitative PCR analysis indicated that glucose (100 mM) and palmitic acid (0.5 mM) treatments suppressed the expression of miR29c significantly (Fig. 2). Interestingly, treatment with insulin (100 nM) or oleic acid (0.5 mM) did not have such an effect and even slightly induced the expression of miR29c (Fig. 2). These findings suggest that miR29c is an important mediator that responds to energy homeostasis-related signals (i.e., increased blood glucose and saturated fatty acids), contributing to the regulation of energy homeostasis in the development of goose fatty liver.

### *Col3a1, Sgk1* and *Insig1* were predicted and validated as target genes of miR29c

**Fig. 3:**
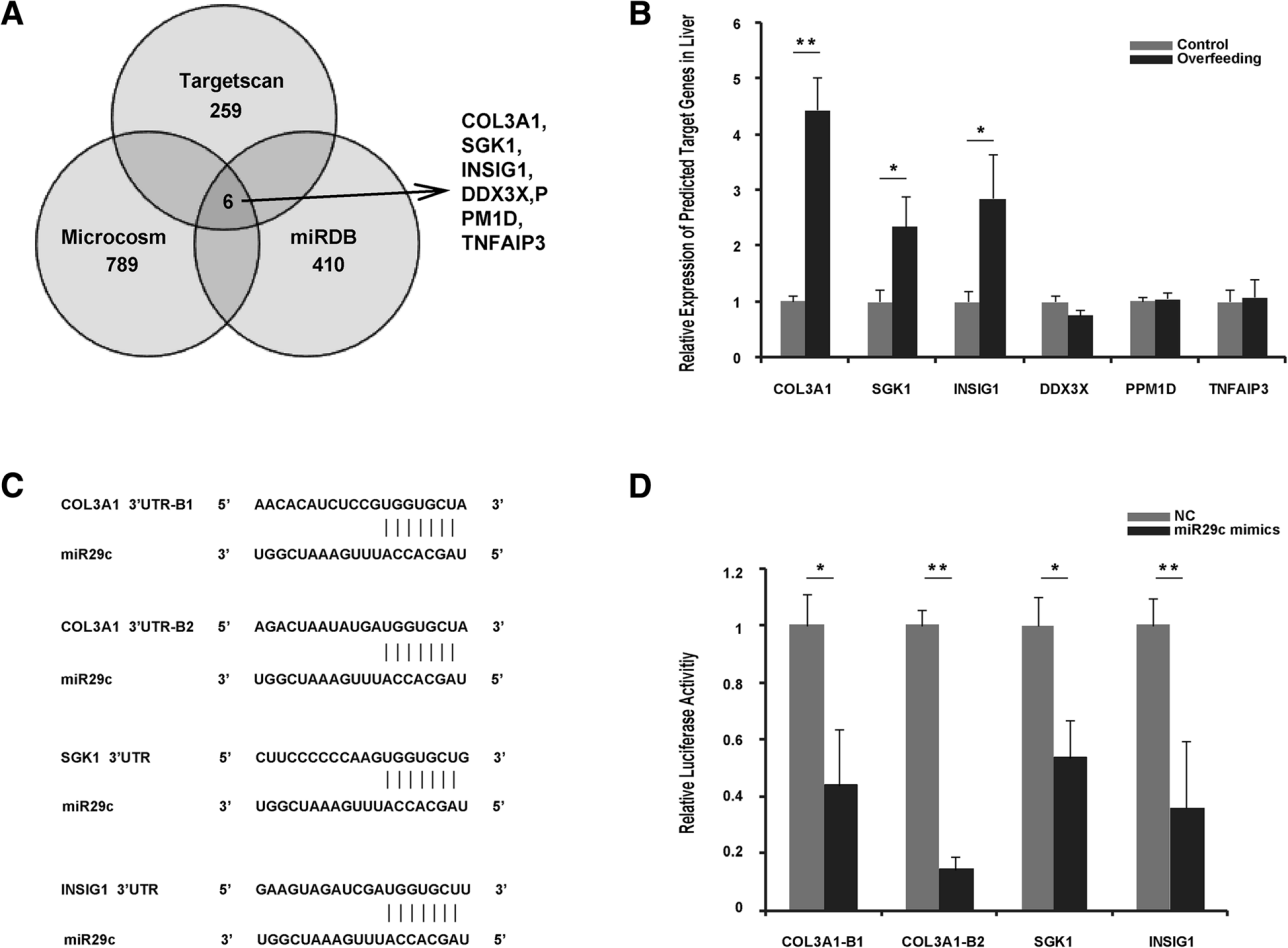
Prediction and validation of goose miR29c. **a** The number of goose miR29c target genes that were predicted by different online software programs. *Col3a1*, *Sgk1*, *Insig1*, DDX3X, PPM1D and TNFAIP3 were identified by multiple databases. **b** Expression of these genes in goose fatty liver vs. normal liver. Expression is presented as the fold change over the control, *n* = 5. **c** The sketch illustrates that the seed sequence of mature miR29c matches the 3’ UTR sequences of goose *Col3a1*, *Sgk1* and *Insig1*. **d** Luciferase activity assay demonstrating that *Col3a1*, *Sgk1* and *Insig1* are the target genes of goose miR29c. Compared to negative control oligonucleotides (NC), miR29c mimics showed significantly reduced relative luciferase activity in CHO cells transfected with the PhRL-TK reporter vector and the pMIR-REPORT luciferase vector containing the 3’ UTR sequence of miR29c target genes, n = 4. All of the data are shown as the means ± SEM. *, ** denote *p* < 0.05, 0.01 vs. control, respectively

Based on the sequence of the mature goose miR29c, we predicted its target genes using three online programs (miRDB, TargetScan and Microcosm). As shown in Fig. 3a, 259, 410 and 789 target genes were predicted by TargetScan, miRDB and Microcosm, respectively. A total of 6 genes, including *Col3a1*, *Sgk1*, *Insig1*, *Ddx3x*, *Ppm1d* and *Tnfaip3*, were common among the predicted genes. To confirm this prediction, the mRNA expression of these genes was determined in goose fatty liver vs. control liver. Corresponding to the suppression of miR29c, the expression of *Col3a1*, *Sgk1* and *Insig1* was significantly induced in goose fatty liver (while the other three genes were not significantly influenced by overfeeding, Fig. 3b). The results suggested that *Col3a1*, *Sgk1* and *Insig1* were most likely to be the target genes of miR29c in the development of goose fatty liver, which was supported by the complete match of the 3’ UTR sequences of the genes to the core sequence of goose mature miR29c (note: *Col3a1* had two matches, Fig. 3c).

To further verify the target genes, expression vectors were constructed that contained the 3’ UTR sequences of goose *Col3a1*, *Sgk1* or *Insig1* plus a fluorescence reporter sequence. When the vectors were co-transfected with miR29c mimics or negative controls into CHO cells, the fluorescence intensity indicating expression of the genes was significantly lower in cells transfected with the miR29c mimics compared to those transfected with the negative control vectors (Fig. 3d, the related data are shown in Additional file 1: Table S1). This result confirmed that *Col3a1*, *Sgk1* and *Insig1* were the target genes of miR29c.

### Palmitate and glucose regulation of the expression of *Sgk1* and *Insig1* is mediated by miR29c

**Fig. 4:**
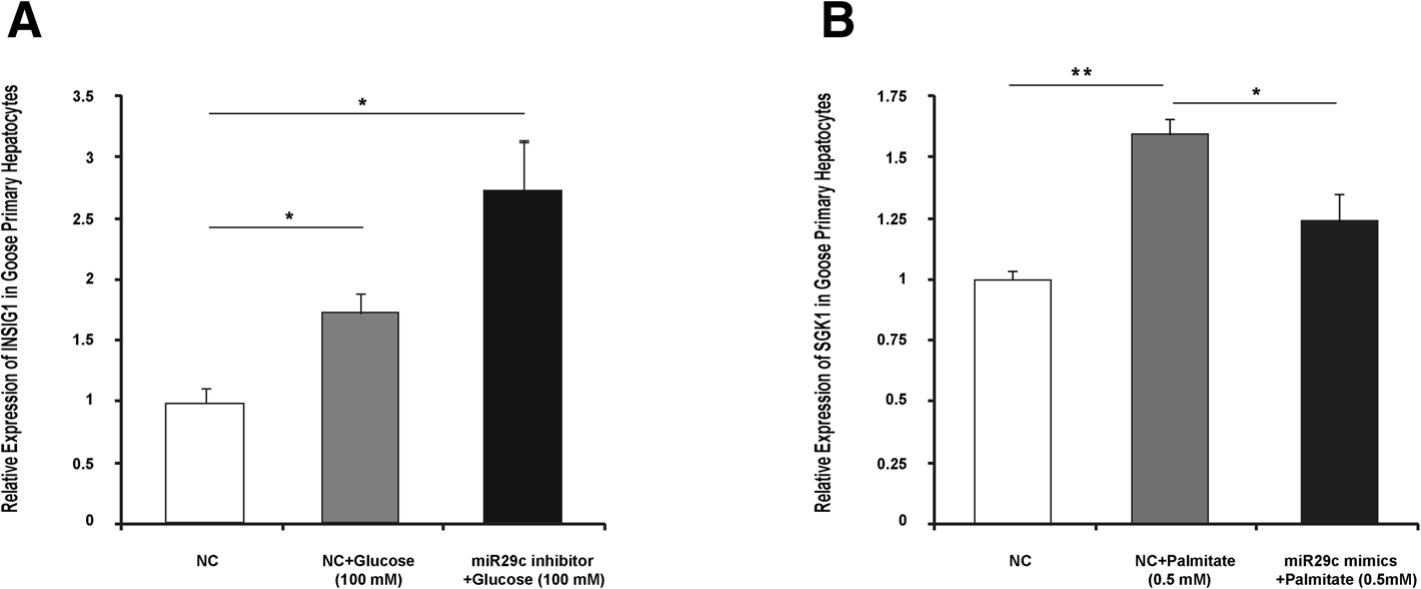
miR29c mediates palmitate/glucose regulation of the expression of its target genes. **a** Glucose induction of *Insig1* is mediated by miR29c in goose primary hepatocytes. **b** Palmitate induction of *Sgk1* is mediated by miR29c in goose primary hepatocytes, *n* = 4. *, ** denote *p* < 0.05, 0.01 vs. control, respectively. All of data are shown as the means ± SEM

Because both palmitate and glucose suppressed the expression of miR29c in goose primary hepatocytes (Fig. 2), we speculated that these factors could regulate the expression of *Sgk1* and *Insig1* via miR29c. Indeed, induction of Sgk1 by palmitate was suppressed by miR29c mimics in goose primary hepatocytes (Fig. 4a), while induction of Insig1 by glucose was further promoted by a miR29c inhibitor (Fig. 4b). These results suggest that miR29c mediates palmitate/glucose regulation of the expression of its target genes.

### RFX1 is a potential transcription factor of miR29c

**Fig. 5:**
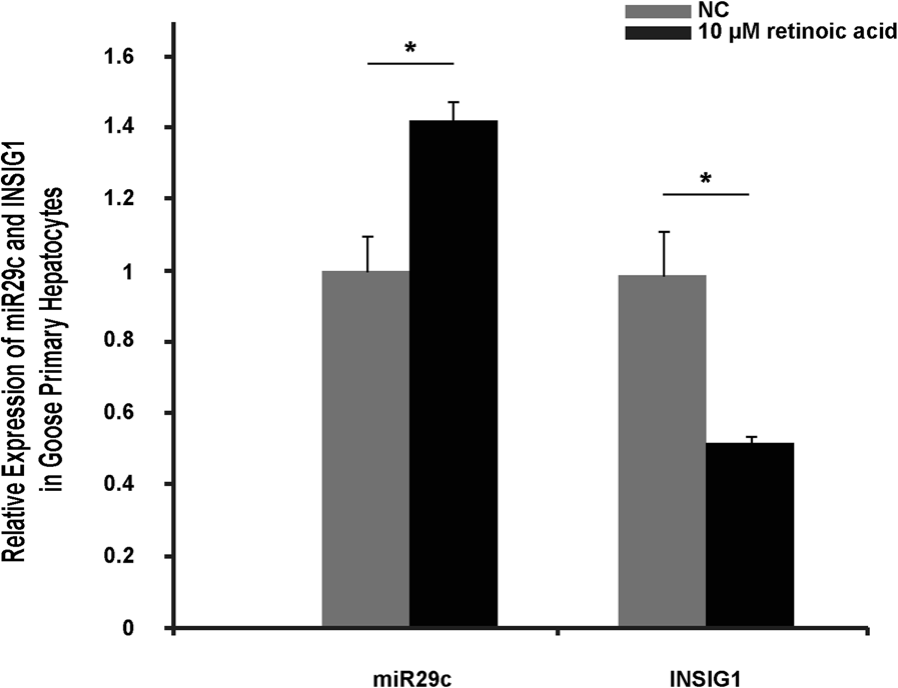
Expression of miR29c and *Insig1* is regulated by retinoic acid in goose primary hepatocytes. The expression level was determined by qRT-PCR analysis using total RNA samples isolated from goose primary hepatocytes treated with or without 10 μM retinoic acid, a drug that regulates the activity of the transcription factor RFX1. The expression level is presented as the fold change over the control, *n* = 4. * denotes *p* < 0.05 vs. control. All of the data are shown as the means ± SEM

To reveal how miR29c is regulated, we tried to identify transcription factors that could bind to the upstream sequence of goose miR29c. For this purpose, primers were designed to amplify the upstream regulatory sequence of goose miR29c using a chicken genomic sequence. Sequence analysis indicated that the upstream sequence of goose miR29c (863 bp in length) was successfully acquired and the goose sequence was similar to the chicken sequence (Additional file 2: Figure S1). Using the goose sequence and online software (Gene Regulation), we predicted that FOXD3, HLF and RFX1 were potential transcription factors of miR29c (Table 1). Among these genes, RFX1 was investigated further because its activity can be regulated by a hepatic steatosis/lipid metabolism-associated factor, retinoic acid (an active metabolite of vitamin A that has been implicated in the regulation of lipid metabolism and hepatic steatosis in animal models) [13, 14]. To verify whether RFX1 was a transcription factor of miR29c, goose primary hepatocytes were treated with/without 10 μM retinoic acid for 24 h. Quantitative PCR analysis indicated that miR29c expression was significantly increased in cells treated with vs. without retinoic acid (Fig. 5). Interestingly, induction of miR29c by retinoic acid was accompanied by suppression of *Insig1* (Fig. 5). These results suggested that RFX1 was a transcription factor of miR29c in goose hepatocytes.

**Table 1:** Prediction of transcription factors based on the goose miR29c upstream sequence^a^

Name	Position (strand)	Core match	Matrix match	Sequence on the (+) strand
*HLF*	134(−)	1	0.936	atttGTAAC
*RFX1*	285(+)	0.98	0.98	aggttgcatgGAAACca
*FOXD3*	581(+)	0.986	0.986	aaTTGTTttttt

^a^The prediction was performed using the ‘Match’ program with default parameters (http://www.gene-regulation.com). The goose miR29c upstream sequence is shown in Additional file 2: Figure S1

## Discussion

Although miR29 family genes play an important role in multiple cellular processes and are involved in the occurrence of several diseases, it is unclear what the function of miR29 is or the mechanism by which it functions in the development of fatty liver. In this study, we reported that expression of miR29c, a widely studied miR29 family gene, was suppressed in energy homeostasis-related tissues (including the liver, adipose tissue and muscle) of overfed geese. Moreover, the *Insig1, Sgk1* and *Col3a1* genes were the targets of miR29c as was predicted by three online programs and validated by in vivo and *vitro* studies. Importantly, palmitate and glucose, factors that can modulate energy homeostasis, suppressed miR29c expression but also induced the expression of *Sgk1* and *Insig1* in goose primary hepatocytes, and the regulation of the target genes by palmitate and glucose was mediated by miR29c. In addition, RFX1 was found to be a potential transcription factor of miR29c. Together, these findings suggest that suppression of miR29c in energy homeostasis related tissues, as a response to hyperglycemia and hyperlipidemia, may play a part in goose fatty liver by modulating energy metabolism via its target genes, *Insig1, Sgk1* and *Col3a1*.

Previous studies have shown that *Sgk1* can stimulate intestinal glucose absorption as well as cellular glucose uptake from blood circulation into relevant tissues [15, 16], and thus, it plays a critical role in glucose transport. Moreover, *Insig1*, another target gene of miR29c, is an important integrator of nutrient and hormonal signals that regulate cholesterol metabolism, lipogenesis, and glucose homeostasis [17]. *Insig1* can bind to SREBP cleavage activating protein (SCAP) and facilitate SREBP retention in the endoplasmic reticulum, thus inhibiting SREBP activation [18] and lipogenesis [19]. Based on the functions of *Sgk1* and *Insig1* in glucose transport and lipogenesis, we infer that the suppression of miR29c and induction of target genes in the liver, muscle and adipose tissue may alleviate the harmful effects of hyperglycemia and severe steatosis by promoting the transportation of blood glucose into these tissues and preventing lipogenesis at the end of the overfeeding period. In addition, it is noteworthy that miR29c targeting to *Insig1* has been shown by a previous report, indicating that overexpressed miR29 profoundly inhibits *Insig1* expression and lipogenesis in rats [6].

miR29c may also be involved in tissue growth during the development of goose fatty liver. Our previous transcriptome analysis has shown that goose fatty liver undergoes dramatic cell growth and proliferation [7]. Since *Col3a1*, a target gene of miR29c, encodes collagen alpha-1(III) chain, a precursor of collagen III [20], and collagen alpha-1(III) acts as the ‘cement’ of tissues (connecting the cells), induction of *Col3a1* may meet the requirements for inducing a dramatic enlargement of goose fatty liver. Moreover, it has been reported that *Sgk1* can regulate cell volume [21] and cell proliferation [22] as well as inhibit apoptosis [23], and *Insig1* plays a role in the growth and differentiation of tissues related to metabolic control [24]. We thus infer that suppression of miR29c in goose fatty liver may provide the goose liver excellent tolerance to severe steatosis by promoting cell growth at the end of the overfeeding period.

However, it is intriguing that suppression of miR29c in goose fatty liver contradicts the induction of miR29c in the muscle, fat and liver of the diabetic rat [6], despite the fact that both geese with fatty livers and rats with diabetes develop IR, the key player in the occurrence of multiple metabolic diseases [2, 6, 10, 25]. This difference is probably due to genetic differences between geese and humans (or other mammals). Based on the functions of *Col3a1*, *Sgk1* and *Insig1* in cell growth, we conclude that miR29c may participate in tissue growth during the development of goose fatty liver, which enables the goose liver to tolerate severe steatosis at the end of the overfeeding period.

There is a large genetic distance between birds and mammals. Many novel genes were generated during the evolution of birds and mammals, and the promoter sequences of some existing genes are different between these species. Additionally, goose fatty liver is physiological. The development of fatty liver in the goose is an evolutional adaption to long-distance migration, and geese may have developed a protective mechanism that prevents the harmful effects of hepatic steatosis. By contrast, human fatty liver is pathological. Therefore, these differences derived from evolution could lead to different mechanisms by which miR29c expression is regulated.

The second possible reason for the difference in miR29c expression between goose and mammals is that miR29c could be subjected to different epigenetic controls. Human metabolic diseases often undergo long-term development, while goose fatty liver only undergoes short-term development. The different periods of time for disease development may lead to different epigenetic controls.

The last possible reason for the difference is that there are other factors in addition to hyperglycemia, hyperinsulinemia and hyperlipidemia that significantly influence miR29c expression in human diseases. In summary, this study demonstrated the uniqueness of the goose fatty liver model, which may provide new ideas for treating human fatty liver disease.

## Conclusions

This study indicated that miR29c was comprehensively suppressed in energy homeostasis-related tissues (the liver, adipose tissue and muscle) of overfed geese. This suppression was partially due to hyperglycemia and hyperlipidemia. *Col3a1*, *Sgk1* and *Insig1* were the targets of miR29c. Expression of the target genes was increased in goose fatty liver vs. control. Regulation of *Sgk1* and *Insig1* by glucose or palmitate was mediated by miR29c. RFX1 is a potential transcription factor of miR29c. Overall, miR29c may play a role in goose fatty liver by modulating energy homeostasis and tissue growth via its target genes, *Col3a1*, *Sgk1* and *Insig1*.

## Methods

### Geese and tissue sampling

A total of 10 healthy 65-day-old Landes geese with the same genetic background raised under the same husbandry conditions were purchased from Rui Nong Farm (Yangzhou, China). The geese were randomly divided into a control group and overfeeding group (*n* = 5, respectively). Five-day-long pre-overfeeding was performed to prepare the overfeeding group of geese for formal overfeeding, which lasted 19 days. The overfeeding procedure and diet regimens were performed as previously described [4]. The geese were euthanized by the intravenous injection with pentobarbital sodium solution (100 mg/kg) before decapitation. Liver, abdominal fat and pectoral muscle samples were taken from geese at sacrifice and were snap frozen in liquid nitrogen and stored at − 70 °C. For isolation of goose primary hepatocytes, embryos at 23 days of hatch were decapitated with a pair of scissors before harvesting their livers.

### Prediction and validation of miR29c target genes

The sequence of goose miR29c was obtained from goose liver transcriptome analysis. This sequence was used for the prediction of the miR29c target genes using three online programs (miRDB, TargetScan and Microcosm) as previously described [26]. The coding sequence (CDS) and 3′ untranslated region (UTR) of the predicted target genes were amplified by PCR with primers designed based on their mRNA sequences (Table 2), followed by sequencing analysis. The identity of the target genes was verified by sequence alignment against homologous sequences from other animal species. The 3’ UTR sequence was used for experimental validation of the miR29c target genes according to previously described procedures [26]. In brief, the 3’ UTR sequence of the target gene was inserted into the multiple cloning site of the pMIR-REPORT luciferase miRNA expression vector (provided by Dr. Zhiliang Gu at the Changshu Institute of Technology, Jiangsu, China). The PhRL-TK reporter vector was used as an internal control to normalize variability due to differences in cell viability and the transfection efficiency. The PhRL-TK reporter vector and pMIR-REPORT luciferase vector containing the 3’ UTR sequences of the miR29c target genes were co-transfected with miR29c mimics (CGAUUUCAAAUGGUGCUAUU) and control oligonucleotides into CHO cells. The relationship between miR29c and its predicted target genes was indicated by the normalized luciferase activity.

**Table 2:** Primers used in this study

Name	Targeted region	Sequence (5′ → 3′)	Size (bp)
*Col3a1*-B1-SacI-F *Col3a1*-B1-Hind III -R	3’ UTR of *Col3a1*–1	cgagctcAACAAAATTTCTGGGGGAAAA cccaagcttCTGGGAGCAATCAGTGCTTT	225
*Col3a1*-B2-SacI-F *Col3a1*-B2-Hind III -R	3’ UTR of *Col3a1*–2	cgagctcTCCTGTCATTGCTGGTCAAG cccaagcttCATGCCAATGACAATCTTTGA	180
*Insig1*-SacI-F *Insig1*-HindIII -R	3’ UTR of *Insig1*	cgagctcTGCCCAAAGGAAGTAGATCG cccaagcttACCCCCTCCACTGCTAAAGT	222
*Sgk1*-SpeI-F *Sgk1*-MluI-R	3’ UTR of *Sgk1*	cgactagtTTTGTTCTTCCCCCAAAGTG cgacgcgtCACACTCTGCAACAGGGAAA	227
*Sgk1*-F *Sgk1*-R	*Sgk1* CDS	CCCCCTTTTAACCCAAATGT GGCATATGAGAAGCCCAAAA	165
*Col3a1*-F *Col3a1*-R	*Col3a1* CDS	AGGCTGAAGGAAACAGCAAA GTCCACGCCAAATTCTTGAT	176
*Insig1*-F *Insig1*-R	*Insig1* CDS	CTGGGTCTCTGGTGGACATT GGAAGCCAAGAACGGATGTA	155
*Ppm1d* -F *Ppm1d* -R	PPM1D CDS	GGCATATGAGAAGCCCAAAA CCACGACTGAATGCTTCTGA	227
*Ddx3x* -F *Ddx3x* -R	DDX3X CDS	GAAAGTTTGCATACCGTTCCA CAGTCCAATCTTTCCCCTCTC	155
*Tnfaip3*-F *Tnfaip3*-R	TNFAIP3 CDS	GCAGATTCGAGGATTTGAGC TTGGGTAGGTTGCCTTCATC	169
*Gapdh*-F *G*a*pdh*-R	GAPDH CDS	GCCATCAATGATCCCTTCAT CTGGGGTCACGCTCCTG	155
F-miR-29c-F F-miR-29c-R	miR29c up**s**tream sequence	ATTACAGCGTGGGAGGAGAC AGAGACGTTTTCCTGGCTCA	863

### Acquisition of the goose miR29c upstream sequence and transcription factor prediction

The upstream sequence of the goose miR29c was amplified and sequenced with primers (Table 2) that were designed according to the upstream sequence of chicken miR29c. The sequence is shown in Additional file 2: Figure S1. Transcription factor prediction was performed using the ‘Match’ program with default parameters (http://www.gene-regulation.com).

### Preparation and treatment of goose primary hepatocytes

Goose primary hepatocytes were isolated from Landes goose embryos at 23 days as previously described [4]. The isolated primary hepatocytes (1 × 10^6^ per well) were cultured in 12-well dishes at 38 °C and 5% CO_2_. For the palmitate treatment, cells were treated with culture media containing 2% bovine serum albumin (BSA) or 2% BSA conjugated to 0.5 mM potassium palmitate for 14 h as previously described [3]. Similarly, for the oleate treatment, cells were treated with or without 0.5 mM sodium oleate for 14 h. For the glucose (Cat. No. G7021, Sigma, USA) and insulin (Cat. No. I5500, Sigma, USA) treatments, cells were treated with culture media with or without 100 mM glucose and 100 nM insulin, respectively. For the retinoic acid treatment, cells were treated with culture media containing 10 μM retinoic acid (Cat. No. R2625-50MG, Sigma, USA) or vehicle (ethanol) for 24 h. For treatment with miR29c mimics, miR29c inhibitors or negative control oligonucleotides (synthesized by GenePharma Co., Ltd., Shanghai, China), oligonucleotides were transfected into goose primary hepatocytes using Lipofectamine 2000 as previously described [26]. In brief, 1.2 μL of Lipofectamine 2000 Reagent was added to 50 μL of serum-free medium and incubated at room temperature for 5 min to make solution A, while 1 μL of pMIR-REPORT-3’UTR, 1 μL of miR-29c mimics/miR-29c mimics NC and 1 μL of PhRL-TK were added to 50 μL of serum-free medium to make solution B. After solution A and B were mixed and incubated at room temperature for 30 min, 100 μL of the complex was added to each well. After treatment, all of the primary hepatocytes were rinsed with PBS twice, followed by harvesting of the cells with 1 mL of TRIzol Reagent (Cat. No. 15596026, Life, USA) per well.

### RNA isolation, cDNA synthesis and qRT-PCR

Total RNA was extracted from tissue samples or cells, and cDNA was synthesized with the purified RNA samples without contamination of genomic DNA, which was removed by DNase I (D2215, TaKaRa Bio, Inc., China) as previously described [3]. The abundance of miR29c was determined by qRT-PCR using primers that were commercially designed based on the sequence of goose mature miR29c. Briefly, PCR amplification was conducted as follows: 94 °C for 2 min, 40 cycles of 94 °C for 20 s, 60 °C annealing for 34 s. The U6 gene was used as an internal control for normalization. The mRNA abundance of the target gene was determined by qRT-PCR as previously described [27]. Briefly, PCR amplification was conducted as follows: 95 °C for 5 min, 40 cycles of 95 °C for 10 s, and 60 °C annealing for 34 s. The goose glyceraldehyde-3-phosphate dehydrogenase gene (GAPDH) gene was used as an internal control. The sequences of the primers used for qRT-PCR analysis are listed in Table 2. The cycle threshold (Ct) was determined with the supplied software. mRNA abundance was calculated using 2^-ΔΔCt^ and is presented as the fold change over the control [28].

### Statistical analysis

All values are expressed as the means ± SEM. Statistical significance was determined by unpaired Student’s *t*-test for two-group comparisons or one-way analysis of variance (ANOVA) for multiple comparisons (Duncan test). *p* < 0.05 was chosen a priori as indicative of statistical significance.

## Additional files

Additional file 1: **Table S1.** Data related to Fig. 3d^*^. (DOCX 14 kb)

Additional file 2: **Figure S1.** The upstream sequence of goose miR29c. (**A**) The sequence of the amplified fragment. (**B**) The picture of the amplified fragment. The sequence was acquired from PCR-based amplification with primers (Table 1) designed based on the chicken sequence and following sequencing analysis. (DOCX 624 kb)

## Abbreviations

BSA: Bovine serum albumin
CDS: The coding sequence
Ct: Cycle threshold
miR29c: miRNA-29c
NAFLD: Nonalcoholic fatty liver disease
UTR: 3′ untranslated region
