# Supplementary material for: Role of miR29c in goose fatty liver is mediated by its target genes that are involved in energy homeostasis and cell growth
Source: BMC Vet Res. 2018 Nov 6;14:325. doi: 10.1186/s12917-018-1653-3 (PMC6219092; doi:10.1186/s12917-018-1653-3)
Supplement: Supplementary file 1 — Table S1. Data related to Fig. 3d*. (DOCX 14 kb) [file 12917_2018_1653_MOESM1_ESM.docx]

**Additional file 1: Table S1.** Data related to Fig. 3D ^*^

| Target Genes | COL3A1-B1 | COL3A1-B2 | SGK1 | INSIG1 |
| --- | --- | --- | --- | --- |
| Negative Control | 41.73±6.69 | 205.00±16.53 | 28.99±4.13 | 63.95±8.77 |
| miR-29c Mimics | 18.4±5.15 | 30.01±1.97 | 15.57±2.92 | 23.06±7.63 |

^*^ The data refer to relative luciferase activities in CHO cells transfected with the PhRL-TK reporter vector and the pMIR-REPORT luciferase vector. All of the data are shown as the means ± SEM.
