# Supplementary material for: Role of miR29c in goose fatty liver is mediated by its target genes that are involved in energy homeostasis and cell growth
Source: BMC Vet Res. 2018 Nov 6;14:325. doi: 10.1186/s12917-018-1653-3 (PMC6219092; doi:10.1186/s12917-018-1653-3)
Supplement: Supplementary file 2 — Figure S1. The upstream sequence of goose miR29c. (A) The sequence of the amplified fragment. (B) The picture of the amplified fragment. The sequence was acquired from PCR-based amplification with primers (Table 1) designed based on the chicken sequence and following sequencing analysis. (DOCX 624 kb) [file 12917_2018_1653_MOESM2_ESM.docx]

**Additional file 2: Figure S1.**


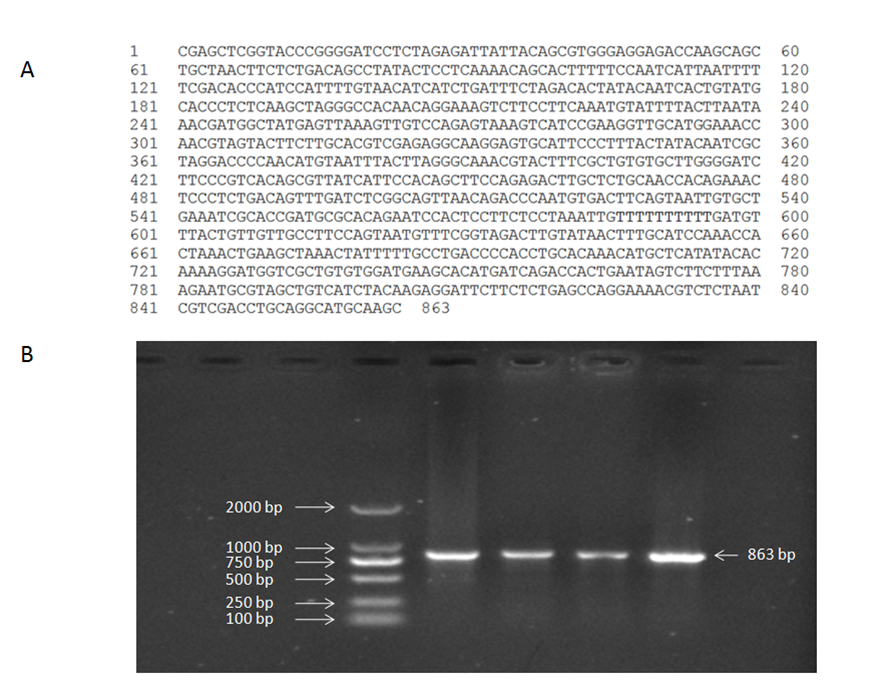


**Fig. S1** The upstream sequence of goose miR29c. (**A**) The sequence of the amplified fragment. (**B**) The picture of the amplified fragment. The sequence was acquired from PCR-based amplification with primers (Table 1) designed based on the chicken sequence and following sequencing analysis.
